# Crystallography in the 21st century^1^

**DOI:** 10.1107/S2052252515017509

**Published:** 2015-11-01

**Authors:** S. Samar Hasnain

**Affiliations:** a Max Perutz Professor of Molecular Biophysics at the University of Liverpool and Editor-in-Chief of IUCr Journals, Barkla X-ray Laboratory of Biophysics, Institute of Integrative Biology, Life Sciences Building, Liverpool L69 7ZB, UK

**Keywords:** cryoEM, crystallography, editorial, GPCR, ribosome, serial crystallography, synchrotron radiation, XFELs

## Abstract

The field of crystallography, which has had a major impact on the sciences in the last 100 years, is continuing to expand scientific horizons as technical and conceptual boundaries are overcome. Structure–function–dynamics will become an integrated theme for many studies as will obtaining structures without the ‘benevolent tyranny’ of crystals.

The year 2015 marks the centenary of the award of the Nobel Prize in Physics to William Henry and William Lawrence Bragg ‘for their services in the analysis of crystal structure by means of X-rays’. In celebrating the centennial of this award, it is useful to reflect on the achievements of a century ago. In the brief but astonishingly productive period after the discovery of X-rays by Röntgen (1895 ▸), we saw Barkla’s recognition that X-rays had light-like properties and thus must scatter (Barkla, 1904 ▸, 1906 ▸, 1908*a* ▸,*b* ▸), followed by Laue’s understanding of how X-rays would diffract from the regular stacking planes of a crystal (Friedrich *et al.*, 1912 ▸), and the Braggs’ application of this to make the first determinations of crystal and, subsequently, molecular structures (Bragg, 1913 ▸; Bragg & Bragg, 1913 ▸). Nobel prizes also came in quick succession with awards to Röntgen (1901), von Laue (1914), the Braggs (1915) and Barkla (1917) (no Nobel prizes were awarded in 1916).

These achievements established the field of X-ray crystallography and highlighted the importance of this route for providing the atomic details necessary for understanding chemical and biological systems and their mechanisms.

The impact of these breakthroughs at the start of the 20th century is apparent in many of the most striking scientific advances of the rest of the century. The nature of the chemical bond; chemical reaction mechanisms; elastic, thermal, magnetic and optical properties of materials; the basis of the genetic code; the relation between protein structure and function; the functioning of biological molecules *in vivo* and new strategies for drug design: all were informed by the elucidation of atomic level structure primarily by diffraction-based methods, using X-rays, electrons and neutrons.

Even though these studies have been central to many discoveries across a wide canvas of sciences, crystallography in the 21st century is in danger of being defined by its historical achievements, and of becoming a victim of its own success. Today, in the minds of many, ‘crystallography’ is perceived as an analytical technique that merely provides structures. Rigorous courses in crystallography are beginning to disappear from many curriculums. Chairs of crystallography are often not being replaced, and new Chairs in the subject have become rare, particularly in non-biological disciplines. Powerful X-ray sources from in-vacuum undulators on ‘diffraction-limited storage rings’ (see *e.g.* Eriksson *et al.*, 2014 ▸), coupled with user-friendly algorithms and highly precise instrumentation with automated control systems, have made the determination of accurate molecular structures a ‘routine’ occurrence in many cases. The resolution at which complex biological structures can be determined is constantly improving – the number of protein structures that are now known at subatomic resolution (*i.e.* ≤1 Å) exceeds 600. The complexity of structures that are being determined today appears to see no bounds, with the structures of the ribosome and G-protein coupled receptors claiming Nobel Prizes in Chemistry in 2009 and 2012, respectively.

It is important for the wider communities of chemists, biochemists, biologists, materials scientists and others who are the ultimate beneficiaries of structure-informed science to appreciate that structure determination should not be perceived as ‘routine’ science. Crystallography needs to meet new challenges as they emerge: some of these we can anticipate, others will emerge as new technical and conceptual boundaries are overcome, requiring the same level of intellectual prowess that made crystallography such a resounding success over the last 100 years. X-ray free-electron lasers (XFELs), serial crystallography, coherent diffractive imaging and electron cryomicroscopy (cryoEM) are already offering challenges, and overcoming them would create new opportunities for a whole range of new and novel science. Harnessing the diffuse-scattering information in diffraction data from biological complexes to provide dynamic information, and bringing *R* factors of protein structures to levels similar to those for small molecules through better modelling, remain an immediate challenge. It is more important now than ever that our field attracts the best minds with interests in chemistry, materials science and biology so that the most complex and challenging problems can be tackled across the sciences. The Editors and staff of IUCr Journals are committed to doing their utmost to bring these developments to the pages of our journals in an efficient, timely, attractive and prominent manner.

Crystallography is by its nature inclusive and always evolving. It has a deep relationship with symmetry, from the sixfold shape of snowflakes described by Kepler in 1611 to today’s interpretation of quasicrystals as projected symmetry from a higher-dimensional space. It is a subject that delights in every new opportunity to explore the structure of matter. Crystallography is no longer just about structure determination (if it ever was), and it is no longer just about the study of crystals (if it ever was). The very definition of a crystal has been revised to accommodate quasicrystals, and may need to be revised again (Senechal, 2015 ▸). Questions such as ‘what is a crystal?’ or ‘what are the limits of crystallography?’ are appropriate questions to pose and address in the columns of our journals. Crystallography has become a multi-dimensional subject in a very literal sense: not only are higher spatial dimensions needed to characterize incommensurate periodicities; not only are one-dimensional and two-dimensional surface properties crucial to our understanding of nanomaterials and crystal growth; but also with the advent of serial femtosecond crystallography (Spence *et al.*, 2012 ▸; Spence, 2015 ▸; Schlichting, 2015 ▸), dynamic processes have brought the temporal dimension firmly into play. X-ray structures at subatomic resolution, combined with high-resolution neutron structures (Blakeley *et al.*, 2015 ▸), allow us to put quantum theory to the test and provide understanding of protein structure–function relationships at electronic levels.

The International Union of Crystallography (IUCr), which became a Scientific Union on 7 April 1947, currently has over 20 Commissions, addressing different realms of the physical, chemical, material and biological sciences, and different experimental techniques and probes. The number of Commissions continues to grow as the outreach of the subject continues to widen. Since the publication of its first peer-reviewed journal, *Acta Crystallographica*, in March 1948, the portfolio of IUCr Journals has continued to expand, responding to new and emerging fields. In March 1968, the Union launched *Journal of Applied Crystallography*, and in October 1994 it published its first peer-reviewed issue of *Journal of Synchrotron Radiation*. Last year, the International Year of Crystallography, saw a major reshaping of all of our journals, preparing them for the coming decades of crystallography-based science and the associated developments in enabling technologies and methods. In its 67th year, the Union finally launched a journal carrying its name, **IUCrJ**. This journal has the simple objective of attracting high-quality science papers of broad scientific significance from across the full breadth of the scientific communities that use results obtained from diffraction or complementary methods. With its distinguished Editorial and Advisory Boards, its rapid production schedule, and open-access publication model, **IUCrJ** is ideally placed to become the natural home for reporting significant advances and new discoveries across the sciences. It has already published significant contributions in the fields of crystal engineering (for example, see Mukherjee *et al.*, 2014 ▸), structural biology (for example, see Frank *et al.*, 2014 ▸) and materials sciences (for example, see Bish *et al.*, 2014 ▸). The question for the community remains: can **IUCrJ** become for the IUCr what *PNAS* is for the NAS? Is the community ready to report its best science in its own journal whose aim is to provide wide scope and high impact? The journal has just launched a cryoEM subsection under the guidance of Richard Henderson (Advisory Board Member), and Werner Kuhlbrandt and Sriram Subramaniam (Co-editors). We encourage the cryoEM community to make the journal their natural home, sharing advances and results with the widest community of structural scientists.

All the IUCr Journals are refereed and published to the highest of standards. All take an efficient and effective approach to the archiving and distribution of supporting data. All offer either full or hybrid open-access publication to allow the widest possible dissemination of research results. An overriding principle is to provide a fair review process that helps authors to communicate their particular scientific advances efficiently and effectively. All our journals are there to communicate scientific advances and technological and methodological breakthroughs. We urge authors to help us strengthen the crystallographic community by reporting significant advances in our journals. We encourage the chemical and materials sciences communities to submit papers to *Structural Science, Crystal Engineering and Materials* (*Acta B*), *Structural Chemistry* (*Acta C*) and *Crystallographic Communications* (*Acta E*). Likewise, we encourage biological communities to send papers reporting significant advances to *Structural Biology* (the new subtitle of *Acta D* from January 2016) and *Structural Biology Communications* (*Acta F*). All these communities, as well as all the other science communities covered by the IUCr Commissions, are welcome to submit papers that aim to report results and discoveries of the widest general interest and implications to **IUCrJ**. *Acta A* will continue to build on the founding goals of *Acta Crystallographica* in terms of reporting *Foundations and Advances* of crystallographic approaches and applications, with its *Advances* section setting a new gold standard for breakthrough publications (Billinge & Miao, 2015 ▸).

Crystallography in the 21st century has progressively relaxed the need for translational symmetry in the specimen under examination. Pair distribution function analysis provides an increasing understanding of defects in crystalline materials, of intermediate-range order as exhibited in nanoparticles and nanocrystallinity, and of short-range order in glasses and liquids. NMR spectroscopy provides insights into the conformation of proteins in solution that are complementary to the determination of a protein structure in a crystal. CryoEM is providing exquisite structures of complex systems at high resolutions. XFEL science is moving towards single-particle structures, fulfilling the dream of obtaining structures (and molecular transforms from individual molecules) without the ‘benevolent tyranny’ of crystals (Johnson & Blundell, 1999 ▸). Structure–function–dynamics is becoming an integrated theme. All of these and many other exciting developments that will shape the expanding field of crystallography for the remainder of the century will find a natural home in IUCr Journals.
